# Prognostic Significance of the Density and Spatial Distribution of Tumor‐Associated Macrophages in Giant Cell Tumor of Bone and Their Association With Denosumab Treatment Responsiveness

**DOI:** 10.1002/mco2.70419

**Published:** 2025-10-20

**Authors:** Yi‐Fan Yang, Jing‐Ru Liu, Ying‐Song Han, Guo‐Qiang Zhu, Hua‐Qing Niu, Bo‐Yu Zheng, Xin Tang, Jian Li, Yi‐Jun Kang, Jin‐Ming Yu, Bo‐Wen Zheng, Bin Zhou

**Affiliations:** ^1^ Department of Orthopaedics Surgery West China Hospital Sichuan University Chengdu China; ^2^ Department of Spine Surgery The Second Xiangya Hospital Central South University Changsha China; ^3^ Department of Oncology Renmin Hospital of Wuhan University Wuhan China; ^4^ Department of Spine Surgery Xiangtan Central Hospital Xiangtan China; ^5^ Department of Orthopedics Surgery Xiangya Hospital Central South University Changsha China; ^6^ Department of Ophthalmology The Second Affiliated Hospital of Zhengzhou University Zhengzhou China; ^7^ Department of Orthopedics Surgery General Hospital of the Central Theater Command Wuhan China; ^8^ Department of Musculoskeletal Tumor Peking University People's Hospital Peking University Beijing China; ^9^ Department of Orthopedics Wuhan Union Hospital, Tongji Medical College, Huazhong University of Science and Technology Wuhan China; ^10^ Department of Spine Surgery The First Affiliated Hospital University of South China Hengyang China

**Keywords:** denosumab, giant cell tumor of bone, polychromatic fluorescence immunohistochemistry, tumor associated macrophages

## Abstract

Given the lack of reliable indicators for predicting prognosis and treatment response in giant cell tumor of bone (GCTB) patients, this study aimed to identify new prognostic factors by analyzing the effect of tumor‐associated macrophages (TAMs) on prognosis and denosumab treatment responsiveness. The expression of CD68⁺TAMs, CD163⁺TAMs, and IRF8⁺TAMs was detected using polychromatic fluorescence immunohistochemistry in 162 GCTB samples. TAM density was quantified through computer‐aided image analysis, and spatial parameters, including nearest neighbor distance (NND) and effective percentage (EP), were measured using HALO software. Results showed that higher densities of CD68⁺ and CD163⁺ TAMs were significantly associated with inferior progression‐free survival (PFS). A smaller NND was linked to shorter PFS. Additionally, higher CD68⁺ EP was associated with poorer PFS, whereas higher CD163⁺ EP correlated with better PFS. Receiver operating characteristic curve analysis demonstrated that TAM parameters' predictive performance was comparable to Campanacci and surgical approach in three subgroups. The ineffective denosumab‐treated group had significantly higher TAMs EP compared to the effective group. In conclusion, TAMs significantly influence the prognosis of GCTB patients and are correlated with certain invasive tumor phenotypes. Elevated TAMs levels may be associated with reduced efficacy of denosumab treatment.

## Graphical abstract

From January 2015 to December 2020, 393 samples of GCTB were collected from five centers.

The density of CD68+ TAMs, CD163+ TAMs, and IRF8+ TAMs was counted by computer‐aided image analysis, and their spatial parameters including NND and EP were measured by HALO. Higher CD68+ TAMs and CD163+ TAMs densities were significantly correlated with inferior PFS. TAMs NND displayed a positive correlation with PFS. Higher CD68+ EP was linked to poorer PFS, whereas higher CD163+ EP was associated with better PFS. The ineffective denosumab‐treated group exhibited significantly higher CD68+ and IRF8+ TAMs density, as well as higher TAMs EP compared to the effective group.

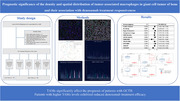

## 1 Introduction

Giant cell tumor of bone (GCTB) constitutes approximately 5% of all bone tumors [1, 2, 3]. It is a rare and locally aggressive osteolytic neoplasm. Complete resection remains the primary treatment for GCTB [4]. However, the high recurrence rate persists due to challenges in achieving complete resection [5, 6]. Denosumab, a RANKL inhibitor, is the most commonly used drug for GCTB. While it can induce favorable initial tumor responses, it may increase the recurrence rate after curettage and trigger tumor sarcomatoid transformation [7]. Traditional chemotherapy is often ineffective for GCTB patients, and radiotherapy increases the risk of malignant transformation [5]. Due to the high recurrence rate of GCTB, identifying new prognostic factors could help guide treatment decisions.

Currently, reliable indicators for predicting the prognosis and treatment response in GCTB patients are lacking. Although the Campanacci and Enneking staging systems are commonly used for clinical guidance and prognostic stratification, substantial variations in prognosis persist even within the same stage [8, 9]. Several studies have identified molecular markers associated with prognosis in GCTB patients [10]. However, these studies primarily focus on the molecular level, which may result in inaccurate prognostic predictions. Tumor‐associated macrophages (TAMs) are key components of the tumor microenvironment, known for their interactions with cancer cells. CD68 is a pan‐macrophage marker that labels TAMs. Studies have shown that its higher density in non‐small cell lung cancer and renal cancer is associated with poor prognosis in tumor tissues [11, 12]. In tumor tissues, TAMs often polarize toward the M2 macrophage phenotype, which stimulate neovascularization, enhance resistance to radiation, and promote tumor invasion and metastasis [13]. CD163 is a selective cell surface marker for M2‐type TAMs [14]. Previous studies have shown that the number of CD163+ TAMs is inversely associated with survival in patients with gastric cancer, breast cancer, and glioma [15, 16, 17]. The expression of interferon regulatory factor 8 (IRF8) in TAMs can induce CTL exhaustion and promote tumor progression, acting as a critical marker of the immunosuppressive microenvironment [18]. IRF8+ TAMs have been associated with higher tumor grades and poorer outcomes [19]. Therefore, this study aims to evaluate these three critical TAM markers.

Additionally, spatial distribution metrics, such as nearest neighbor distance (NND) and effective percentage (EP), provide valuable insights into tumor invasion potential and prognosis prediction [20]. Previous studies have shown that M2‐TAMs can directly affect cancer cells through interactions with adjacent secretory cells, paracrine signaling, and metabolic factors [21]. Therefore, characterizing the spatial distribution of TAMs is crucial for fully understanding their relationship with clinical prognosis.

The tumor immune microenvironment (TIME) plays a critical role in tumor development. Existing literature indicates that immune cells in the microenvironment can serve as prognostic markers for GCTB [10]. Although the long‐term outcomes of GCTB treatment with denosumab remain debated, it is still regarded as one of the most effective therapies [22]. However, the impact of TAM density and spatial distribution on the clinical‐pathological characteristics, prognosis, and denosumab treatment response in GCTB patients remains unclear. This study aims to clarify these relationships and establish the clinical significance of TAMs. We hypothesize that TAM density and spatial distribution may influence GCTB prognosis and affect the therapeutic efficacy of denosumab.

## 2 Results

### 2.1 Patient Flow and Characteristics

**FIGURE 1:**
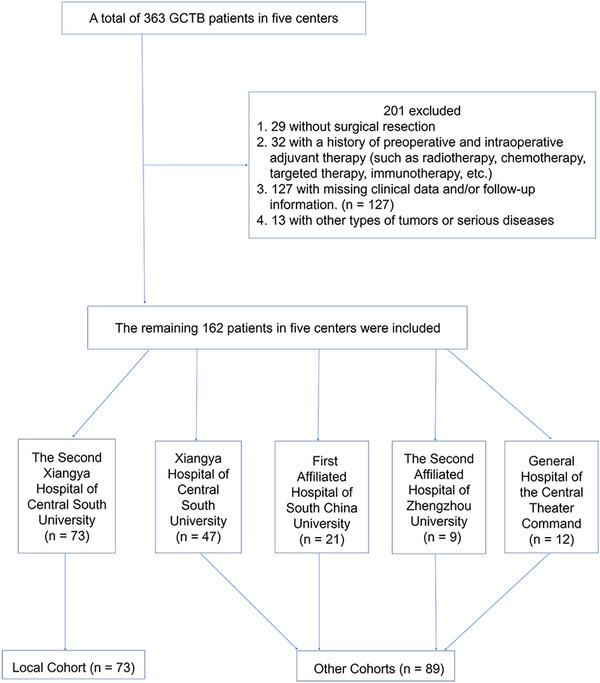
The flow of patients’ inclusion.

The study initially identified 363 patients with GCTB recorded in the electronic medical record systems of five medical centers between 2015 and 2020. Of these, 29 patients had not undergone surgical resection, 32 had received preoperative or intraoperative adjuvant therapies, including radiotherapy, chemotherapy, targeted therapy, or immunotherapy, 127 had incomplete clinical data or lacked follow‐up information, and 13 had concurrent diagnoses of other malignancies or severe comorbidities. After applying these exclusion criteria, a total of 162 patients were ultimately included in the study cohort (Figure 1). The median follow‐up time for progression‐free survival (PFS) in the 162 patients was 62 months (range, 5–205 months). In the recurrence group, the median time to local recurrence was 52.5 months (range, 7–107 months).

**FIGURE 2:**
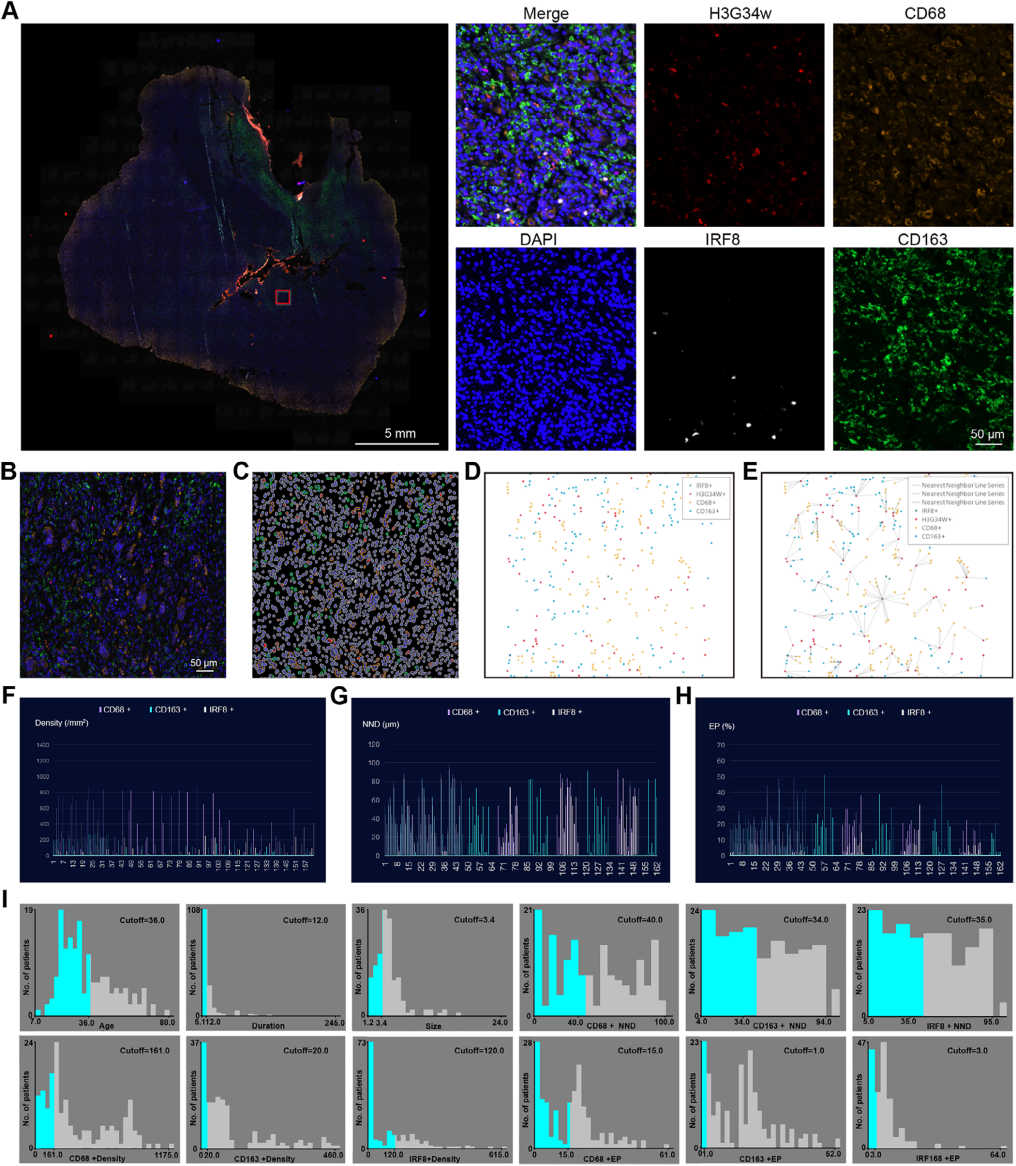
Halo software was used to analyzing each patient's multiplex immunohistochemistry images. (A) Representative images of multiplex immunohistochemistry. Blue, nucleus; red, tumor cells; white, IRF8+ TAMs; yellow, CD68+ TAMs; green, CD163+ TAMs. (B) Spatial map of TAMs and tumor cells generated by Halo software. (C) The projection of original image. (D) Spatial plot generated by Halo. (E) Nearest line series was generated by Halo for measuring NND and EP. (F–H) TAMs parameters of every patient included in this study. (I) The best cutoffs of every continuous variables generated by X‐tile.

Table 1 presents demographic and clinicopathological data of the study cohort. The study cohort had an average age of 35.4 years, with 93 (57.4%) males and 69 (42.6%) females. Fifty‐four (33.3%) GCTB samples were located in the axial skeleton, and 108 (66.7%) GCTB samples were located in the extra‐axial skeleton. The average tumor length was 4.72 ± 2.3 cm, and the mean duration of preoperative symptoms was 17.2 ± 25.5 months. Preoperative neurological dysfunction was present in 34 (21.0%) patients, while postoperative neurological dysfunction was observed in 50 (62.3%) patients. By the end of the follow‐up period in December 2021, there were 64 (39.5%) cases of recurrence. After analyzing each patient's multiplex immunohistochemistry images using Halo software (Figure 2A–E), the average densities of CD68⁺ TAMs, CD163⁺ TAMs, and IRF⁺ TAMs were 357.0 ± 293.5/mm^2^, 100.4 ± 123.6/mm^2^, and 101.4 ± 118.7/mm^2^, respectively. The mean NND was 188.9 ± 150.6, 42.5 ± 27.7, and 43.3 ± 27.0 µm, respectively. The EP was 30.13 ± 17.98%, 15.7 ± 11.8%, and 7.4 ± 7.6%, respectively. Additionally, we summarized the density and spatial distribution parameters of TAMs for each patient, as shown in Figure 2F–H. For survival analysis, patients were stratified into low and high groups based on the optimal cutoff values for TAMs density, NND, and EP, as determined by X‐tile software (Figure 2I).

**TABLE 1:** Demographic and clinicopathological data in the study.

Characteristic	Number of patients (%)
Age (years)	35.4 ± 14.6
Gender	
Male	93 (57.4)
Female	69 (42.6)
Tumor size (cm)	4.72 ± 2.3
Duration of symptoms (months)	17.2 ± 25.5
Preoperative neurological dysfunction	
No	128 (79.0)
Yes	34 (21.0)
Tumor location	
Axial	54 (33.3)
Extra‐axial	108 (66.7)
Postoperative neurological dysfunction	
No	112 (37.7)
Yes	50 (62.3)
Recurrence during follow‐up	
No	98 (60.5)
Yes	64 (39.5)
Campanacci stage	
I	14 (8.6)
II	55 (34.0)
III	93 (57.4)
Enneking	
Intracopartmental	86 (53.1)
Extracopartmental	76 (46.9)
Type of resection	
EA	90 (55.6)
EI	72 (44.4)
CD68^+^ Density (cells/mm2)	357.0 ± 293.5
CD163^+^ Density (cells/mm2)	100.4 ±123.6
IRF8^+^ Density (cells/mm2)	101.4 ± 118.7
CD68^+^ NND (µm)	188.9 ± 150.6
CD163^+^ NND (µm)	42.5 ± 27.7
IRF8^+^ NND (µm)	43.3 ± 27.0
CD68^+^ EP (%)	15.77±12.32
CD163^+^ EP (%)	15.7 ± 11.8
IRF8^+^ EP (%)	7.4 ± 7.6
PFS (months)	66.27 ± 39.79

Abbreviations: EA, Enneking appropriate; EI, Enneking inappropriate; EP, effective percentage; NND, nearest neighbor distance; PFS, progression‐free survival.

### 2.2 Influence of TAMs Parameters on the PFS of GCTB Patients

**FIGURE 3:**
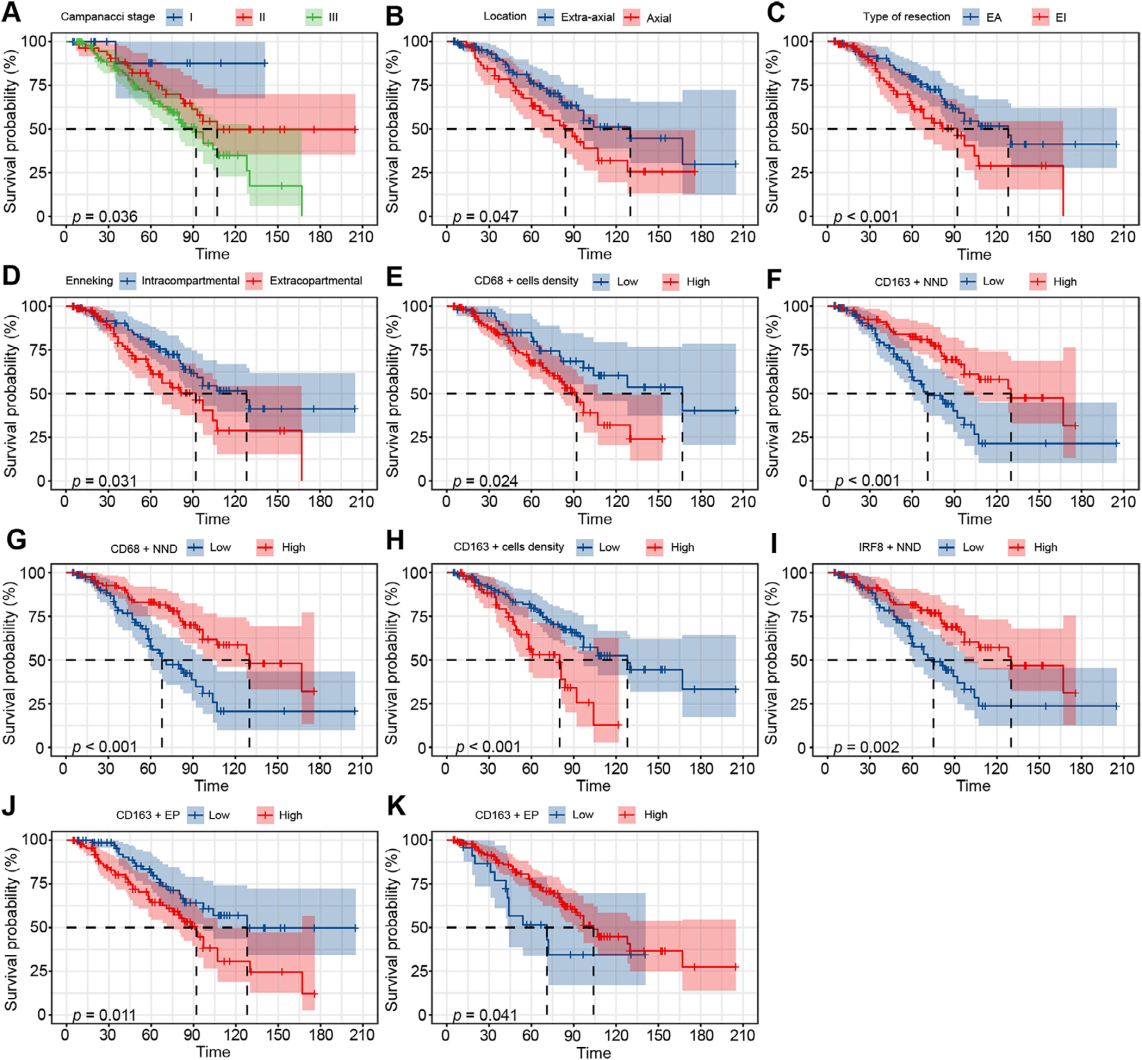
Positive results of univariate analysis of the relationship between risk factors and PFS. (A) Higher Campanacci, (B) tumors in axial, (C) EI resection, and (D) extracopartmental Enneking were significantly correlated with inferior PFS. (E and F) Higher densities of CD68+ TAMs and CD163+ TAMs were significantly correlated with inferior PFS (*p* = 0.024 and 0.001). (G–I) TAMs NND showed a positive correlation with PFS. (J) Higher CD68+ EP was associated with poorer PFS, while (K) higher CD163+ EP was associated with better PFS.

Higher densities of CD68+ TAMs and CD163+ TAMs were significantly correlated with inferior PFS (*p* = 0.024 and 0.001). TAMs NND showed a positive correlation with PFS, suggesting that a smaller NND was associated with a shorter PFS. CD68+ EP and CD163+ EP of TAMs were significantly related to PFS. Higher CD68+ EP was associated with poorer PFS, while higher CD163+ EP was associated with better PFS (Table 2, Figure 3). We also identified significant associations between axial tumor location, Enneking appropriate (EA) operative method, Campanacci stage, and Enneking stage and PFS. Higher Campanacci stage was associated with worse PFS, and the extra‐compartmental type in the Enneking stage showed poorer PFS than the intra‐compartmental type (Table 2, Figure 3). Multivariate Cox regression analysis, following backward stepwise selection based on the Akaike information criterion (AIC), identified tumor location (HR = 2.03, 95% CI: [1.20–3.44], *p* = 0.008), operative method (HR = 2.27, 95% CI: [1.00–5.12], *p* = 0.049), preoperative Campanacci stage (HR = 1.79, 95% CI: [1.09–2.95], *p* = 0.022), density of CD163+ TAMs (HR = 2.09, 95% CI: [1.19–3.67], *p* = 0.010), CD68+ NND (HR = 0.39, 95% CI: [0.22–0.69], *p* = 0.001), CD68+ EP (HR = 2.48, 95% CI: [1.39–4.41], *p* = 0.002), CD163+ EP (HR = 0.35, 95% CI: [0.17–0.71], *p* = 0.003), and IRF8+ EP (HR = 0.54, 95% CI: [0.30–0.96], *p* = 0.036) as independent predictors of PFS (Table 2).

**TABLE 2:** Univariate and multivariate analysis of relationship between risk factors and PFS.

Factors	Categories	Mean PFS	Number of patients	Univariate analysis		Multivariate analysis	
				*Χ* ^2^	*p*‐value	HR (95% CI)	*p*
Age (years)	Young (≤ 36.0)	63.95 ± 39.04	101	2.467	0.116		
	Old (> 36.0)	70.10 ± 41.03	61				
Gender	Female	64.61 ± 38.50	69	0.017	0.897		
	Male	67.49 ± 40.88	93				
Tumor location	Axial	66.11 ± 40.05	54	3.960	**0.047**	2.03 (1.20–3.44)	**0.008**
	Extra‐axial	66.57 ± 39.63	108				
Duration of symptoms (months)	Short (≤ 12.0)	63.98 ± 38.99	102	1.417	0.234		
	Long (> 12.0)	70.15 ± 41.15	60				
Preoperative neurological dysfunction	No	66.63 ± 41.91	128	0.050	0.723		
	Yes	64.91 ± 31.03	34				
Postoperative neurological dysfunction	No	67.75 ± 42.69	112	3.667	0.056		
	Yes	63.38 ± 32.58	50				
Tumor size (cm)	Small (≤ 3.4)	71.73 ± 41.51	51	2.235	0.135		
	Large (> 3.4)	63.76 ± 38.91	111				
Type of resection	EA	72.88 ± 42.45	86	12.242	**<0.001**	2.27 (1.00–5.12)	**0.049**
	EI	58.00 ± 36.21	76				
Campanacci stage	I	76.96 ± 45.26	14	6.647	**0.036**	1.79 (1.09–2.95)	**0.022**
	II	61.97 ± 34.81	55				
	III	52.79 ± 41.33	93				
Enneking	Intracopartmental	76.74 ± 40.48	86	4.642	**0.031**	0.51 (0.22–1.17)	0.112
	Extracopartmental	54.41 ± 35.69	76				
CD68^+^ density (cells/mm^2^)	Low (≤ 161.0)	80.35 ± 48.21	51	5.114	**0.024**		
	High (> 161.0)	59.79 ± 33.52	111				
CD163^+^ density (cells/mm^2^)	Low (≤ 20.0)	75.02 ± 42.69	105	11.340	**<0.001**	2.09 (1.19–3.67)	**0.010**
	High (> 20.0)	50.14 ± 27.55	57				
IRF8^+^ density (cells/mm^2^)	Low (≤ 120.0)	62.48 ± 39.59	40	1.205	0.272		
	High (> 120.0)	67.51 ± 39.94	122				
CD68^+^ NND (µm)	Low (≤ 40.0)	55.19 ± 39.95	77	12.684	**<0.001**	0.39 (0.22–0.69)	**0.001**
	High (> 40.0)	76.29 ± 40.63	85				
CD163^+^ NND (µm)	Low (≤ 34.0)	54.51 ± 39.64	81	11.139	**<0.001**		
	High (> 34.0)	78.02 ± 40.44	81				
IRF8^+^ NND (µm)	Low (≤35.0)	56.05 ± 36.32	78	9.184	**0.002**		
	High (> 35.0)	75.75 ± 40.72	84				
CD68^+^ EP (%)	Low (≤ 15.0)	73.38 ± 40.15	72	6.519	**0.011**	2.48 (1.39–4.41)	**0.002**
	High (> 15.0)	60.58 ± 37.94	90				
CD163^+^ EP (%)	Low (≤ 1.0)	55.17 ± 34.03	23	4.195	**0.041**	0.35 (0.17–0.71)	**0.003**
	High (> 1.0)	68.10 ± 40.48	139				
IRF8^+^ EP (%)	Low (≤ 3.0)	65.21 ± 40.41	63	2.317	0.128	0.54 (0.30–0.96)	**0.036**
	High (> 3.0)	66.94 ± 38.72	99				

*Note*: Bold values indicate *p* <0.05.

Abbreviations: EA, Enneking appropriate; EI, Enneking inappropriate; EP, effective percentage; NND, nearest neighbor distance; PFS, progression‐free survival.

### 2.3 Relationship Between TAMs Parameters and Clinicopathological Features

Patients with preoperative neurological dysfunction showed elevated CD163+ EP (*p* = 0.048). Operative methods were significantly associated with CD68+ NND, CD163+ NND, and IRF8+ NND, with patients undergoing EA surgery showing longer NND (*p* < 0.001). The preoperative Enneking stage was significantly correlated with the NND of CD68+, CD163+, and IRF8+ TAMs. The extracopartmental growth type had a lower NND than the intracopartmental growth type (*p* < 0.001) (Table S1). CD68+ density and tumor size are significantly positively related (*p* = 0.04), while IRF8+ density and duration of symptoms are significantly positively related (*p* = 0.007) (Table S2). Analysis of correlations between TAM parameters revealed a significant negative correlation between the density of CD68+ and IRF8+ TAMs (*p* < 0.001), with no significant correlations observed between the other density parameters. A significant positive correlation was observed between the NND of different TAM subpopulations (*p* < 0.001), as well as between the EP of different TAM subpopulations (*p* < 0.001) (Table S2). No significant correlations were found between the two spatial distribution parameters within the same TAM subpopulation.

### 2.4 TAMs Parameters in the Denosumab Treatment Group

**FIGURE 4:**
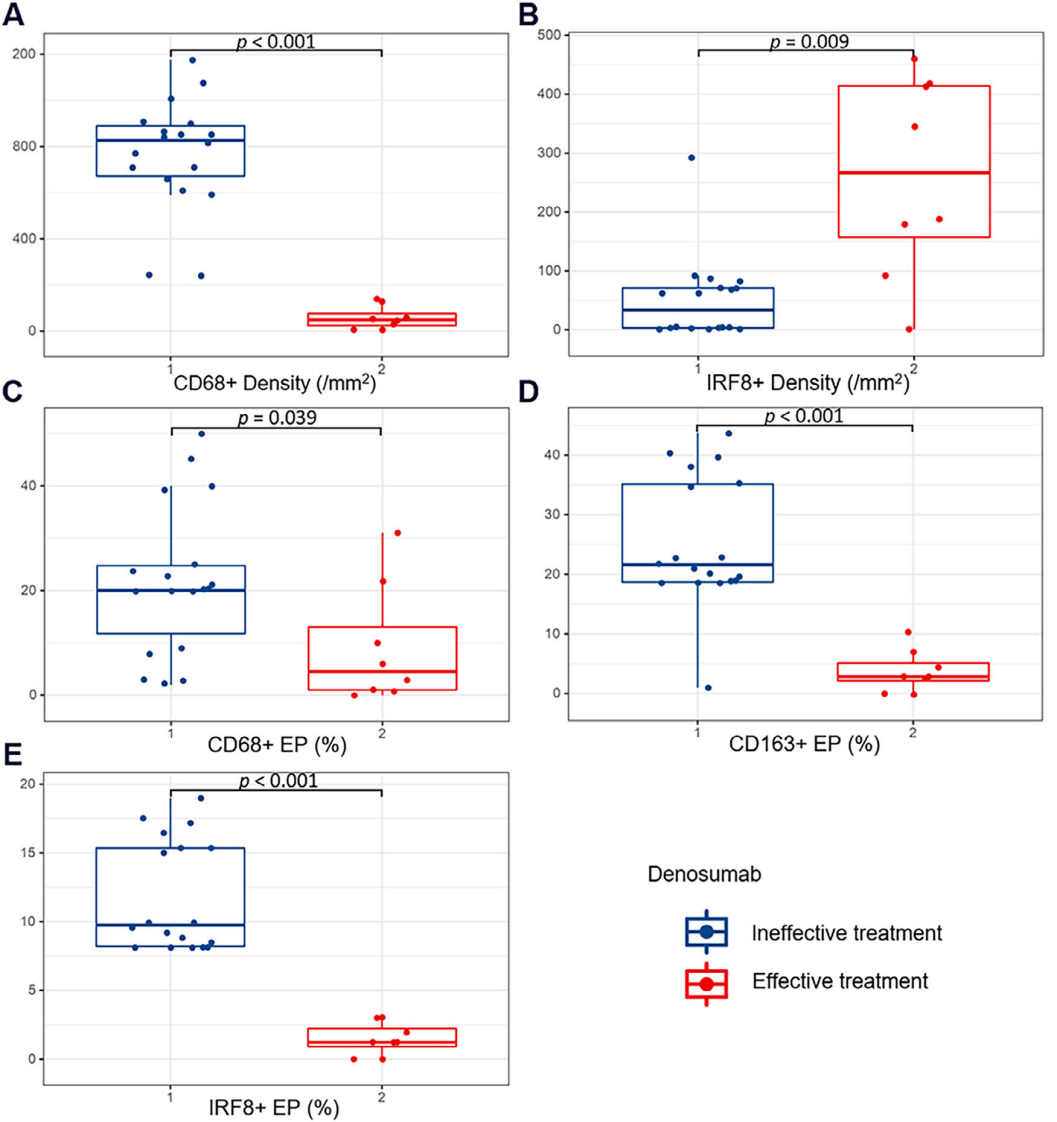
Comparative analysis results with significant differences between tumor‐associated macrophages parameters and denosumab treatment responsiveness. The ineffective group had significantly higher (A) densities of CD68+ (*p* < 0.001) and (B) IRF8+ TAMs (*p* = 0.009), as well as (C–E) higher TAMs EP compared to the effective group (*p* < 0.05).

Among the 26 patients treated with denosumab, eight reported symptom relief, whereas 18 showed no response. The ineffective group had significantly higher densities of CD68+ (*p* < 0.001) and lower densities of IRF8+ TAMs (*p* = 0.009), as well as higher TAMs EP compared to the effective group (*p* < 0.05) (Table 3, Figure 4).

**TABLE 3:** Relationship between TAMs parameters and denosumab treatment responsiveness.

TAMs parameters	Denosumab treatment responsiveness		*p* value
	Ineffective (*n* = 18)	Effective (*n* = 8)	
Density (/mm^2^)			
CD68+	767.7	58.3	**< 0.001**
CD163+	140.0	96.8	0.431
IRF8+	50.6	262.0	**0.009**
NND (µm)			
CD68+	36.1	42.5	0.515
CD163+	30.1	36.5	0.536
IRF8+	31.1	37.5	0.511
EP (%)			
CD68+	21.8	9.3	**0.039**
CD163+	25.4	3.9	**< 0.001**
IRF8+	11.7	1.4	**< 0.001**

*Note*: Bold values indicate *p* <0.05.

Abbreviations: EA, Enneking appropriate; EI, Enneking inappropriate; EP, effective percentage; NND, nearest neighbor distance; PFS, progression‐free survival; TAMs, tumor‐associated macrophages.

### 2.5 Comparison of the Prognostic Predictive Power of Campanacci Staging, Enneking Staging, and TAM Parameters

Receiver operating characteristic (ROC) curve analysis revealed that the prognostic predictive ability of the densities of the three TAM subpopulations ranked as follows, from highest to lowest: IRF8+ TAMs density, CD163+ TAMs density, and CD68+ TAMs density. The prognostic predictive abilities of the NND ranked as follows, from highest to lowest: CD68+ NND, IRF8+ NND, and CD163+ NND. The prognostic predictive abilities of EP, in descending order, were as follows: CD68+ EP, IRF8+ EP, and CD163+ EP. We compared the best prognostic predictive parameters, including IRF8+ TAMs density, CD68+ NND, and CD68+ EP, with the Campanacci and Enneking stages. The prognostic predictive abilities, ranked from highest to lowest, were as follows: Campanacci stage, CD68+ NND, CD68+ EP, IRF8+ TAMs density, and Enneking stage (Figure S1).

## 3 Discussion

This study revealed significant associations between high TAMs density, close NND, and high EP with factors including poorer recurrence‐free survival, larger tumor volume, preoperative neurological dysfunction, and axial tumor location. Furthermore, tumor location, operative method, preoperative Campanacci staging, CD163+ TAMs density, CD68+ NND, and CD163+ EP were identified as independent predictors of PFS. These findings offer new insights into prognosis stratification for GCTB patients and establish a theoretical foundation for future targeted regulation of TAMs in GCTB treatments.

The prognostic role of TAMs remains debated due to their diverse activation and polarization across different cancers [23]. However, a growing body of evidence suggests that elevated TAM levels in malignancies independently predict poorer patient prognosis and survival, as well as increased resistance to radiotherapy and chemotherapy [24, 25]. In our study, we found a significant correlation between higher TAMs density and shorter PFS, consistent with previous studies in breast cancer, bladder urothelial carcinoma, chondroblastoma, head and neck squamous cell carcinoma, renal clear cell carcinoma, and other tumors [26, 27, 28, 29, 30]. However, the prognostic significance of CD68+ TAMs remains debated, likely because CD68 is a general macrophage marker rather than a specific indicator for tumor‐related TAMs [31]. This may explain the higher density of CD68 compared to CD163 and IRF8 in our study. Recent research confirms that CD163+ TAMs promote colorectal cancer cell migration and invasion via IL‐6, which modulates the STAT3/miR‐506‐3p/FoxQ1 pathway [32]. IRF8+ TAMs were found to induce the depletion of cytotoxic T lymphocytes in tumor tissue, fostering tumor growth, consistent with our observations [18]. Moreover, TAM density was positively correlated with symptom duration and tumor volume. This association is supported by prior literature suggesting that larger tumor volumes are more invasive [33], and high‐density TAMs promote tumor cell invasiveness. TAMs contribute to tumor angiogenesis both directly through Sema4D expression, which acts on plexinB1 receptors in vascular endothelial cells, and indirectly by promoting extracellular matrix degradation via MMPs and cathepsins secretion [34, 35]. The correlation between TAMs and disease duration suggests that TAMs may increase as the disease progresses, a hypothesis that requires further molecular‐level research.

Previous studies have shown that the proximity of immune cells to tumor cells can affect tumor progression and patient prognosis [21, 36]. Therefore, analyzing the spatial distribution of TAMs is essential. In our study, we found that the EP and NND of TAMs were significantly associated with PFS. Close proximity between TAMs and tumor cells, or TAMs within the paracrine signaling range of tumor cells, is thought to exert local effects on tumor cells through direct interactions, paracrine signaling, and metabolic factors, thereby enhancing tumor cell invasiveness [21]. Furthermore, earlier research has shown that a reduced distance between immune cells and tumor cells can improve the therapeutic response to immunotherapy [37]. Notably, our findings showed that higher CD68+ EP was linked to poorer prognosis in GCTB patients, whereas higher CD163+ EP was associated with better prognosis. Therefore, the impact of the number of TAMs in close proximity to tumor cells on tumor invasiveness warrants further investigation. Overall, the spatial distribution parameters of TAMs discussed here may be valuable in predicting the effectiveness of TAM‐targeting immunotherapy and guiding prognosis stratification in the future.

TAMs can express Siglec‐15, an immunoreceptor that interacts with the adapter protein DAP12‐Syk signaling pathway. This interaction regulates the RANKL/RANK‐mediated PI3K, AKT, and ERK signaling pathways during osteoclast formation in vitro, ultimately increasing osteoclast activity [38]. Denosumab, a RANKL inhibitor, suppresses osteoclast activity [39]. Our study discovered that the EP of TAMs in the effective group was significantly lower than in the ineffective group. This suggests that patients with higher TAM levels may have reduced denosumab treatment efficacy. This finding supports the molecular mechanisms mentioned above, providing insights into predicting denosumab's therapeutic response and guiding its clinical application. It also provides a theoretical basis for combining macrophage‐targeting drugs with denosumab. Additionally, denosumab therapy may be considered for patients with lower TAM levels.

This study has several limitations. As a retrospective study, it limits our ability to establish causality. Large‐scale prospective studies are needed to validate our findings. Second, we did not elucidate the molecular mechanisms involved. Further research is required to uncover the precise molecular pathways through which TAMs influence clinical outcomes in GCTB, thus laying the foundation for future TAM‐targeting immunotherapy. Third, in order to reduce interference factors as much as possible and make the analysis results more accurate and rigorous, we set most variables as binary variables, which may make the ROC sensitivity result not so high. Finally, our sample size is small. Given the relatively recent use of denosumab for GCTB, future clinical trials are needed.

## 4 Conclusion

The data in this study indicate that TAMs may significantly affect the prognosis of patients with GCTB and are strongly associated with certain invasive phenotypes of the tumor. TAMs parameters in the three subgroups have good predictive performance, comparable to Campanacci and Enneking staging. Additionally, patients with higher TAMs levels showed reduced denosumab treatment efficacy.

## 5 Materials and Methods

### 5.1 Study Design

This retrospective multicenter study reviewed medical records of GCTB patients from five treatment centers, spanning from January 2015 to December 2020. The exclusion criteria are shown in Figure 1. Some patients were also included in the previous study [1, 2, 3]. All patients underwent surgical treatment, with pathological diagnosis confirmed by at least two pathologists through HE staining and the specific antibody H3.3G34W [1, 40].

### 5.2 Patients and Tissue Sample

Clinical and pathological data were extracted from patients' medical records. Campanacci staging was based on preoperative imaging, categorizing patients into stages I, II, and III [41]. According to the Enneking staging system, patients were classified into intra‐compartmental and extra‐compartmental groups [9]. Tumor location was classified as axial or extra‐axial based on lesion site. The type of surgical resection was assessed by two experienced pathologists using postoperative specimens and classified as EA (surgical margin as recommended by the Enneking Classification) or Enneking inappropriate (EI, surgical margin not recommended by the Enneking Classification) based on the surgical margin [42]. EI resection refers to a resection procedure that does not fully comply with the principles of tumor resection, during which part or all of the tumor may not be completely removed due to technical or other reasons. EA resection refers to surgery that fully complies with tumor resection principles, where the tumor is removed along with a layer of surrounding healthy tissue (i.e., the safety margin) to ensure no residual tumor cells [4]. Denosumab is also administered to patients with large preoperative tumors, extensive tissue infiltration, or incomplete resection during surgery [43]. Complete remission, partial remission, and stable disease were classified into effective groups, and tumor progression was classified into ineffective groups based on the Response Evaluation Criteria in Solid Tumors [44]. GCTB tissue samples were collected from the pathology departments of multiple hospitals. The samples were formalin‐fixed, paraffin‐embedded, and sectioned into 4‐µm thick slices for analysis.

### 5.3 Follow‐Up of Patients

Patients were followed up at regular intervals after surgery. In the first year post‐surgery, follow‐up visits were conducted every 3 months. From the second to the third year, follow‐up visits were scheduled every 6 months, and from the third to the fifth year, they occurred annually. After 5 years post‐surgery, follow‐up visits were conducted every 1–2 years. In cases of patient death, the time of death and the cause were recorded. For surviving patients, imaging examinations were performed to assess their current status. MRI imaging was utilized to detect early signs of tumor recurrence. In cases of recurrence, further surgical intervention was carried out, and the recurrent tumors were resected and confirmed through histopathological examination. The primary outcome variable, PFS, was defined as the duration between tumor resection and the first occurrence of tumor progression, as determined through MRI images and/or pathological examination of resected specimens [45]. For patients without tumor recurrence, the event was recorded as “Censored.”

### 5.4 Multiplex Immunohistochemistry

As previously described [40], quantitative immunofluorescence was performed using the AlphaTSA 5‐color fluorescence staining kit (AlphaTSA, Beijing, China). CD68, CD163, and IRF8 levels were assessed in each tumor tissue using a sequential staining protocol, with simultaneous detection of H3.3G34W and 4′,6‐diamidino‐2‐phenylindole (DAPI; PerkinElmer). Briefly, formalin‐fixed paraffin‐embedded sections were deparaffinized and subjected to antigen retrieval in a pressure cooker with Tris‐ethylenediaminetetraacetic acid buffer (pH 9.0) for 10 min. The sections were incubated overnight with primary antibodies at 4°C, followed by antigen blocking with 3% H_2_O_2_ for 15 min and 10% goat serum for 30 min at room temperature. Horseradish peroxidase (HRP)‐conjugated secondary antibodies were then applied at room temperature for 1 h, followed by tyramide‐based HRP activation at 37°C for 20 min. Residual HRP activation was quenched with 1 mM benzoic hydrazide with 0.15% H_2_O_2_. H3.3G34W detection was performed using goat anti‐mouse HRP and XTSA620, while CD68, CD163, and IRF8 were detected with goat anti‐rabbit HRP and XTSA570, XTSA520, and XTSA690 conjugates, respectively. Finally, coverslips were affixed to the slides with ProLongGold Antifade reagent containing DAPI and left to air‐dry overnight.

### 5.5 Automated Image Analysis

The method previously described was utilized [46]. In brief, TAM‐rich sections were examined using an inverted Nikon Eclipse Ti microscope, and representative area images were captured with a Nikon DS‐Ri 1‐U3 camera and NIS‐Elements AR 3.0 software (Figure 2A). Computer‐assisted image analysis (Image‐Pro Plus 6.0, Media Cybernetics Inc, Rockville, MD) was used to quantify TAMs in 10 areas of interest, with DAPI staining the cell nuclei. The tumor compartment was identified by H3.3G34W positivity, and the stromal compartment was delineated by excluding the tumor mask from the DAPI compartment. TAMs density, representing the average number of positively stained cells per unit area (expressed as cells/mm^2^), was the measured value (Figure 2F). Images were reviewed by human observers, and those with staining artifacts or containing less than 3% tumor tissue were excluded from the analysis.

### 5.6 Cell Spatial Distribution

Spatial coordinates of TAMs and tumor cells were integrated into the spatial map by the HALO software (Figure 2B–E). We utilized the Spatial Analysis Algorithm to compute the average NND between TAMs and their closest tumor cell. Additionally, we calculated the EP of TAMs within a radius range of tumor cells from 0 to 50 mm, defined as the ratio of specific TAM subtypes within a given tumor cell radius to the total corresponding TAMs across the entire tumor section [47, 48]. Subsequently, we derived NND and EP data for further analysis (Figure 2G,H).

### 5.7 Statistical Analysis

Statistical analyses were performed using SPSS version 26.0 (IBM Corp., Armonk, NY, USA) and Python version 3.13.3 (Python Software Foundation, Wilmington, DE, USA). Categorical data were summarized as frequencies and assessed with the chi‐square test. Quantitative data were presented as mean ± standard deviation and analyzed using Student's *t*‐test or one‐way ANOVA. The relationship between continuous variables was evaluated using Pearson's or Spearman's correlation tests. X‐tile software (version 3.6.1) was employed to establish the optimal threshold for survival analysis [49]. Patients were categorized into two subgroups (≤ cutoff or > cutoff) based on this threshold. The threshold represented the point with the lowest *p*‐value from the log‐rank test, with appropriate corrections [49]. Kaplan–Meier analysis was used to examine survival differences between groups, and multivariate Cox proportional hazard models were employed to identify independent factors significantly associated with PFS while enrolling the significant variables (*p* < 0.05) from univariate analysis. Kaplan–Meier analysis was used to compare survival differences between groups. To identify independent prognostic factors for PFS, a multivariate Cox proportional hazards model was fitted using backward stepwise selection based on the AIC. Multicollinearity was assessed using variance inflation factors (VIFs), confirming that all variables retained in the final model had VIF values below 5. ROC curve analysis was used to compare the predictive capabilities of Campanacci, type of resection, TAMs density, and spatial distribution parameters. All tests were two‐sided, with statistical significance set at *p* < 0.05.

## Author Contributions

Y.F.Y., and J.R.L. contributed equally to this study; B.Z., J.M.Y., and B.W.Z. contributed equally to this study; G.Q.Z., H.Q.N., B.Y.Z., and B.W.Z. participated in data acquisition for the multiple cohort research; B.W.Z. did the data analysis and interpretation; B.Z., J.M.Y., X.T., J.L., Y.J.K., and Y.S.H. supervised the research; and Y.F.Y., J.R.L., and B.W.Z. contributed to drafting and revision of the manuscript. All authors read and approved the final manuscript.

## Ethics Statement

The study protocol was approved by the Institutional Review Board of The First Affiliated Hospital, University of South China, Hunan, China (No. 2023324025301). Written informed consent was obtained from each patient for publication of this study.

## Conflicts of Interest

The authors declare no conflicts of interest.

## Supporting information




**Table S1**: Relationship between TAMs parameters and categorical viables of clinicopathological characteristics.
**Table S2**: Correlation analysis of different TAMs parameters and their relation to continuous viables of clinicopathological characteristics.
**Figure S1**:Roc curve of different predictors.
(A): Comparison of the prognostic ability of density of different TAMs subtypes.
CD68^+^Density: (AUC:0.541 95％CI 0.450‐0.631)
CD163^+^Dsentiy: (AUC:0.545 95％CI 0.454‐0.636)
IRF8^+^Density: (AUC:0.562 95％CI 0.473‐0.651)
(B): Comparison of the prognostic ability of NND of different TAMs subtypes.
CD68+NND (AUC:0.585 95％CI 0.495‐0.657)
CD163+NND (AUC:0.565 95％CI 0.474‐0.65)
RF8+NND (AUC:0.567 95％CI 0.477‐0.657)
(C): Comparison of the prognostic ability of EP of different TAMs subtypes.
CD68+EP (AUC:0.583 95％CI 0.494‐0.673)
CD163+EP (AUC:0.538 95％CI 0.446‐0.630)
IRF8+EP (AUC:0.566 95％CI 0.475‐0.657)
(D): Comparison of the prognostic ability of TAMs parameters, type of resection, Campanacci stage and Enneking stage.
Type of resection (AUC:0.513 95％CI 0.421‐0.604)
Campanacci stage (AUC:0.599 95％CI 0.512‐0.686)
IRF8^+^Density (AUC:0.562 95％CI 0.473‐0.651)
CD68^+^NND (AUC:0.585 95％CI 0.495‐0.675)
CD68^+^EP (AUC:0.583 95％CI 0.494‐0.673)

## Data Availability

All data requests relevant to the study can correspond to Dr. Bo‐Wen Zheng (xcbowen@foxmail.com).
