# Supplementary material for: Prognostic Significance of the Density and Spatial Distribution of Tumor‐Associated Macrophages in Giant Cell Tumor of Bone and Their Association With Denosumab Treatment Responsiveness
Source: MedComm (2020). 2025 Oct 20;6(11):e70419. doi: 10.1002/mco2.70419 (PMC12538002; doi:10.1002/mco2.70419)
Supplement: Supplementary file 1 — Table S1: Relationship between TAMs parameters and categorical viables of clinicopathological characteristics. Table S2: Correlation analysis of different TAMs parameters and their relation to continuous viables of clinicopathological characteristics. Figure S1:Roc curve of different predictors. (A): Comparison of the prognostic ability of density of different TAMs subtypes. CD68+Density: (AUC:0.541 95％CI 0.450‐0.631) CD163+Dsentiy: (AUC:0.545 95％CI 0.454‐0.636) IRF8+Density: (AUC:0.562 95％CI 0.473‐0.651) (B): Comparison of the prognostic ability of NND of different TAMs subtypes. CD68+NND (AUC:0.585 95％CI 0.495‐0.657) CD163+NND (AUC:0.565 95％CI 0.474‐0.65) RF8+NND (AUC:0.567 95％CI 0.477‐0.657) (C): Comparison of the prognostic ability of EP of different TAMs subtypes. CD68+EP (AUC:0.583 95％CI 0.494‐0.673) CD163+EP (AUC:0.538 95％CI 0.446‐0.630) IRF8+EP (AUC:0.566 95％CI 0.475‐0.657) (D): Comparison of the prognostic ability of TAMs parameters, type of resection, Campanacci stage and Enneking stage. Type of resection (AUC:0.513 95％CI 0.421‐0.604) Campanacci stage (AUC:0.599 95％CI 0.512‐0.686) IRF8+Density (AUC:0.562 95％CI 0.473‐0.651) CD68+NND (AUC:0.585 95％CI 0.495‐0.675) CD68+EP (AUC:0.583 95％CI 0.494‐0.673) [file MCO2-6-e70419-s001.docx]

**Prognostic significance of the density and spatial distribution of** **tumor-associated macrophages in giant cell tumor of bone and their association with denosumab treatment responsiveness**

Yi-Fan Yang^1,2#^, MD, PhD; Jing-Ru Liu^3#^, MD, PhD; Ying-Song Han^4^, MS; Guo-Qiang Zhu^5^, MD, PhD; Hua-Qing Niu^6^, MD, PhD; Bo-Yu Zheng^7^, MD, PhD; Xin Tang^1^, MD, PhD; Jian Li^1^, MD, PhD; Yi-Jun Kang^2^, MD, PhD; Jin-Ming Yu ^3^ MD, PhD; Bo-Wen Zheng^8,9,10^, MD, PhD; Bin Zhou^2*^, MD, PhD

^1^Department of Orthopaedics Surgery, West China Hospital, Sichuan University, Chengdu 610041, China

^2^Department of Spine Surgery, The Second Xiangya Hospital, Central South University, Changsha 410011, China

^3^Department of Oncology, Renmin Hospital of Wuhan University, Wuhan 430060, China

^4^Department of Spine Surgery, Xiangtan Central Hospital, Xiangtan 411100, China

^5^Department of Orthopedics Surgery, Xiangya Hospital, Central South University, Changsha 410011, China

^6^Department of Ophthalmology, The Second Affiliated Hospital of Zhengzhou University, Zhengzhou 450014, China

^7^Department of Orthopedics Surgery, General Hospital of the Central Theater Command, Wuhan 430061, China

^8^Department of Musculoskeletal Tumor, Peking University People's Hospital, Peking University, Beijing, China

^9^Beijing Key Laboratory of Musculoskeletal Tumor, Peking University People's Hospital, Beijing, China.

^10^Department of Spine Surgery, The First Affiliated Hospital, University of South China, Hengyang 421001, China

**^#^Co-first authors**

^*^**Corresponding author:** Bin Zhou, MD, PhD, Department of Spine Surgery, The Second Xiangya Hospital, Central South University, 139 Renminzhong Road, Changsha, Hunan 410011, China. Tel.: (86)-13607439546.

Email: f2zhoubin@csu.edu.cn

**Table S1**: Relationship between TAMs parameters and categorical viables of clinicopathological characteristics.

| Factors | Categories | Number  of  patients | CD68+Density (/mm^2^) | | CD163+Density (/mm^2^) | | IRF8+Density (/mm^2^) | |
| --- | --- | --- | --- | --- | --- | --- | --- | --- |
|  |  |  | Mean level | P | Mean level | P | Mean level | P |
| Gender | Female | 69 | 375.1±298.3 | 0.503 | 122.1±149.9 | 0.072 | 93.6±127.7 | 0.476 |
|  | Male | 93 | 343.5±294.5 |  | 84.4±98.3 |  | 107.2±112.6 |  |
| Tumor location | Axial | 54 | 416.8±323.9 | 0.085 | 101.5±154.7 | 0.944 | 94.6±109.2 | 0.606 |
|  | Extra-axial | 108 | 327.0±277.1 |  | 99.9±106.2 |  | 104.8±124.1 |  |
| Type of resection | EA | 86 | 358.0±274.6 | 0.963 | 91.7±119.9 | 0.341 | 98.3±106.6 | 0.727 |
|  | EI | 76 | 355.8±319.5 |  | 110.3±128.5 |  | 104.9±132.4 |  |
| Preoperative neurological dysfunction | No | 128 | 336.4±276.9 | 0.139 | 108.8±128.7 | 0.094 | 103.6±121.5 | 0.647 |
|  | Yes | 34 | 434.3±351.2 |  | 68.8±99.7 |  | 93.1±110.9 |  |
| Postoperative neurological dysfunction | No | 112 | 329.4±273.3 | 0.103 | 100.1±140.1 | 0.959 | 99.2±119.7 | 0.721 |
|  | Yes | 50 | 418.6±335.1 |  | 101.2±140.1 |  | 106.4±118.7 |  |
| Campanacci  classification | I | 14 | 332.4±291.3 | 0.214 | 138.9±114.4 | 0.071 | 71.3±77.3 | 0.091 |
|  | II | 55 | 304.7±264.6 |  | 71.0±102.3 |  | 129.1±139.6 |  |
|  | III | 93 | 391.5±311.3 |  | 112.0±134.2 |  | 89.6±108.7 |  |
| Enneking  classification | Intracopartmental | 86 | 358.0±274.6 | 0.963 | 91.7±119.9 | 0.341 | 98.3±106.6 | 0.727 |
|  | Extracopartmental | 76 | 355.8±319.5 |  | 110.3±128.5 |  | 104.9±132.4 |  |

| Factors | Categories | Number  of  patients | CD68+NND (μm) | | CD163+NND (μm) | | IRF8+NND (μm) | |
| --- | --- | --- | --- | --- | --- | --- | --- | --- |
|  |  |  | Mean level | P | Mean level | P | Mean level | P |
| Gender | Female | 69 | 47.8±26.1 | 0.787 | 41.5±26.9 | 0.701 | 43.0±26.1 | 0.885 |
|  | Male | 93 | 48.9±28.0 |  | 43.2±28.7 |  | 43.6±28.3 |  |
| Tumor location | Axial | 54 | 47.5±27.3 | 0.760 | 42.0±27.8 | 0.858 | 42.0±27.9 | 0.670 |
|  | Extra-axial | 108 | 48.9±27.2 |  | 42.8±28.0 |  | 44.0±27.1 |  |
| Type of resection | EA | 86 | 60.3±25.5 | **<0.001** | 54.6±26.6 | **<0.001** | 55.0±25.4 | **<0.001** |
|  | EI | 76 | 35.0±22.4 |  | 28.9±22.6 |  | 30.1±23.1 |  |
| Preoperative neurological dysfunction | No | 128 | 50.0±27.0 | 0.163 | 43.8±27.7 | 0.242 | 45.1±27.2 | 0.115 |
|  | Yes | 34 | 42.6±27.2 |  | 37.5±28.3 |  | 36.8±26.9 |  |
| Postoperative neurological dysfunction | No | 112 | 50.4±27.0 | 0.157 | 44.4±27.8 | 0.189 | 45.4±27.0 | 0.141 |
|  | Yes | 50 | 43.9±27.2 |  | 38.2±27.9 |  | 38.6±27.5 |  |
| Campanacci  classification | I | 14 | 50.0±31.5 | 0.975 | 44.0±32.8 | 0.977 | 45.0±30.6 | 0.970 |
|  | II | 55 | 48.3±26.9 |  | 42.5±27.6 |  | 43.0±27.2 |  |
|  | III | 93 | 48.3±26.9 |  | 42.3±27.6 |  | 43.3±27.1 |  |
| Enneking  classification | Intracopartmental | 86 | 60.3±25.5 | **<0.001** | 54.6±26.6 | **<0.001** | 55.0±25.4 | **<0.001** |
|  | Extracopartmental | 76 | 35.0±22.4 |  | 28.9±22.6 |  | 30.1±23.1 |  |

| Factors | Categories | Number  of  patients | CD68+EP | | CD163+EP | | IRF8+EP | |
| --- | --- | --- | --- | --- | --- | --- | --- | --- |
|  |  |  | Mean level | P | Mean level | P | Mean level | P |
| Gender | Female | 69 | 17.1±13.5 | 0.244 | 16.2±13.4 | 0.626 | 8.6±9.0 | 0.114 |
|  | Male | 93 | 14.8±11.5 |  | 15.2±11.1 |  | 6.6±6.7 |  |
| Tumor location | Axial | 54 | 15.8±12.9 | 0.979 | 15.6±10.1 | 0.978 | 7.7±7.3 | 0.787 |
|  | Extra-axial | 108 | 15.8±11.5 |  | 15.7±13.0 |  | 7.4±8.1 |  |
| Type of resection | EA | 86 | 16.1±12.0 | 0.761 | 15.6±12.1 | 0.920 | 7.2±5.6 | 0.582 |
|  | EI | 76 | 15.5±13.0 |  | 15.8±12.2 |  | 7.8±9.7 |  |
| Preoperative neurological dysfunction | No | 128 | 15.4±11.4 | 0.584 | 14.7±11.8 | **0.048** | 7.0±5.8 | 0.308 |
|  | Yes | 34 | 17.0±15.7 |  | 19.3±12.8 |  | 9.3±12.7 |  |
| Postoperative neurological dysfunction | No | 112 | 15.4±11.3 | 0.587 | 15.0±11.9 | 0.277 | 7.0±6.0 | 0.291 |
|  | Yes | 50 | 16.7±14.7 |  | 17.2±12.5 |  | 8.4±10.8 |  |
| Campanacci  classification | I | 14 | 19.6±14.1 | 0.317 | 13.9±11.3 | 0.317 | 8.4±10.8 | 0.905 |
|  | II | 55 | 16.6±12.8 |  | 14.0±11.6 |  | 7.4±9.0 |  |
|  | III | 93 | 14.7±11.9 |  | 16.9±12.5 |  | 7.4±6.5 |  |
| Enneking  classification | Intracopartmental | 86 | 16.1±12.0 | 0.761 | 15.6±12.1 | 0.920 | 7.2±5.6 | 0.582 |
|  | Extracopartmental | 76 | 15.5±13.0 |  | 15.8±12.2 |  | 7.8±9.7 |  |

Abbreviation: TAMs, tumor-associated macrophages; EA, Enneking appropriate; EI, Enneking inappropriate; NND, nearest neighbor distance; EP, effective percentage; PFS, progression-free survival

| TAMs  Diameters | | CD68^+^  Density | CD163^+^  Density | IRF8^+^  Density | CD68^+^  NND | CD163^+^  NND | IRF8^+^  NND | CD68^+^  EP | CD163^+^  EP | IRF8^+^  EP |
| --- | --- | --- | --- | --- | --- | --- | --- | --- | --- | --- |
| CD68^+^  TAMs  Diameters  Density | RR  RR, P | 1.000 | 0.048 | -0.357 | 0.022 | 0.029 | 0.12 | 0.554 | 0.639 | 0.463 |
|  | P |  | 0.548 | **＜0.001** | 0.782 | 0.714 | 0.882 | **＜0.001** | **＜0.001** | **＜0.001** |
| CD163^+^  Density | RR | 0.048 | 1.000 | 0.006 | 0.003 | 0.009 | -0.004 | -0.024 | 0.054 | -0.052 |
|  | P | 0.548 |  | 0.944 | 0.969 | 0.912 | 0.956 | 0.759 | 0.497 | 0.511 |
| IRF8^+^  Density | RR | -0.357 | 0.006 | 1.000 | -0.110 | -0.095 | -0.112 | -0.219 | -0.283 | -0.212 |
|  | P | **＜0.001** | 0.944 |  | 0.165 | 0.231 | 0.154 | **0.005** | **＜0.001** | **0.007** |
| CD68^+^  NND | RR | 0.022 | 0.003 | -0.110 | 1.000 | 0.985 | 0.984 | 0.126 | -0.058 | -0.032 |
|  | P | 0.782 | 0.969 | 0.165 |  | **＜0.001** | **＜0.001** | 0.110 | 0.460 | 0.683 |
| CD163^+^  NND | RR | 0.029 | 0.009 | -0.095 | 0.985 | 1.000 | 0.939 | 0.138 | -0.062 | -0.037 |
|  | P | 0.714 | 0.912 | 0.231 | **＜0.001** |  | **＜0.001** | 0.080 | 0.436 | 0.636 |
| IRF8^+^  NND | RR | 0.012 | -0.004 | -0.112 | 0.984 | 0.939 | 1.000 | 0.108 | -0.054 | -0.027 |
|  | P | 0.882 | 0.956 | 0.154 | **＜0.001** | **＜0.001** |  | 0.170 | 0.492 | 0.734 |
| CD68^+^EP | RR | 0.554 | -0.024 | -0.219 | 0.126 | 0.138 | 0.108 | 1.000 | 0.371 | 0.315 |
|  | P | **＜0.001** | 0.759 | **0.005** | 0.110 | 0.080 | 0.170 |  | **＜0.001** | **＜0.001** |
| CD163^+^EP | RR | 0.639 | 0.054 | -0.283 | -0.058 | -0.062 | -0.054 | 0.371 | 1.000 | 0.440 |
|  | P | **＜0.001** | 0.497 | **＜0.001** | 0.460 | 0.436 | 0.492 | **＜0.001** |  | **＜0.001** |
| IRF8^+^EP | RR | 0.463 | -0.052 | -0.212 | -0.032 | -0.037 | -0.027 | 0.315 | 0.440 | 1.000 |
|  | P | **＜0.001** | 0.511 | **0.007** | 0.683 | 0.636 | 0.734 | **＜0.001** | **＜0.001** |  |
| Age | RR | -0.112 | -0.030 | 0.144 | 0.109 | 0.098 | 0.115 | 0.065 | -0.124 | -0.093 |
|  | P | 0.157 | 0.709 | 0.068 | 0.169 | 0.215 | 0.144 | 0.409 | 0.116 | 0.241 |
| Duration of symptoms | RR | -0.023 | -0.030 | 0.212 | 0.022 | 0.023 | 0.021 | 0.010 | -0.059 | -0.052 |
|  | P | 0.776 | 0.709 | **0.007** | 0.779 | 0.770 | 0.791 | 0.897 | 0.459 | 0.510 |
| Tumor size | RR | 0.161 | 0.102 | -0.063 | -0.068 | -0.061 | -0.072 | 0.108 | 0.107 | 0.119 |
|  | P | **0.040** | 0.198 | 0.428 | 0.389 | 0.444 | 0.362 | 0.170 | 0.177 | 0.132 |

**Table S2**: Correlation analysis of different TAMs parameters and their relation to continuous viables of clinicopathological characteristics.

Abbreviation: TAMs, tumor-associated macrophages; EA, Enneking appropriate; EI, Enneking inappropriate; NND, nearest neighbor distance; EP, effective percentage; PFS, progression-free survival


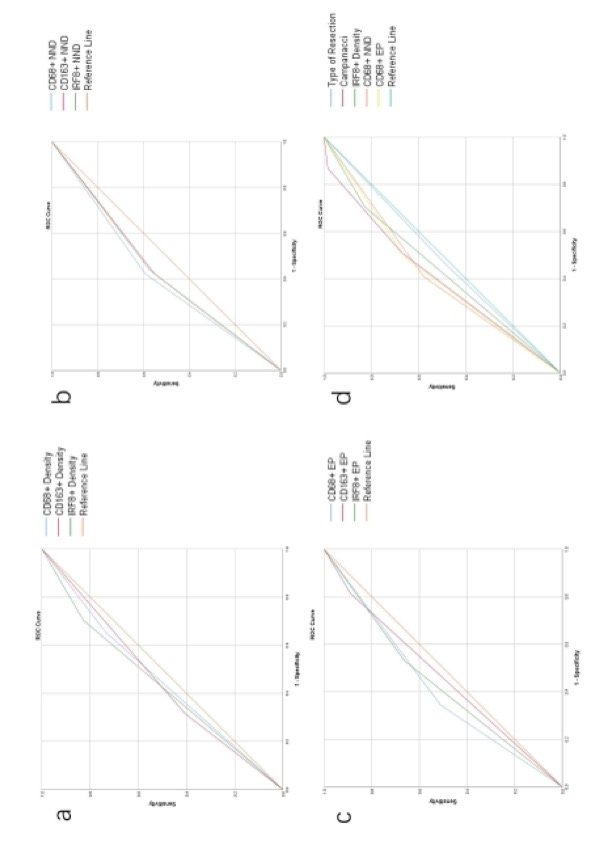


**A**

**B**

**D**

**C**

**Figure S1：**Roc curve of different predictors.

(A): Comparison of the prognostic ability of density of different TAMs subtypes.

CD68^+^Density: (AUC:0.541 95％CI 0.450-0.631)

CD163^+^Dsentiy: (AUC:0.545 95％CI 0.454-0.636)

IRF8^+^Density: (AUC:0.562 95％CI 0.473-0.651)

(B): Comparison of the prognostic ability of NND of different TAMs subtypes.

CD68+NND (AUC:0.585 95％CI 0.495-0.657)

CD163+NND (AUC:0.565 95％CI 0.474-0.65)

RF8+NND (AUC:0.567 95％CI 0.477-0.657)

(C): Comparison of the prognostic ability of EP of different TAMs subtypes.

CD68+EP (AUC:0.583 95％CI 0.494-0.673)

CD163+EP (AUC:0.538 95％CI 0.446-0.630)

IRF8+EP (AUC:0.566 95％CI 0.475-0.657)

(D): Comparison of the prognostic ability of TAMs parameters, type of resection, Campanacci stage and Enneking stage.

Type of resection (AUC:0.513 95％CI 0.421-0.604)

Campanacci stage (AUC:0.599 95％CI 0.512-0.686)

IRF8^+^Density (AUC:0.562 95％CI 0.473-0.651)

CD68^+^NND (AUC:0.585 95％CI 0.495-0.675)

CD68^+^EP (AUC:0.583 95％CI 0.494-0.673)
